# The Dielectric Constant of Ba_6−3*x*_(Sm_1−*y*_Nd_*y*_)_8+2*x*_Ti_18_O_54_ (*x* = 2/3) Ceramics for Microwave Communication by Linear Regression Analysis

**DOI:** 10.3390/ma13245733

**Published:** 2020-12-16

**Authors:** Tiancheng He, Caihuan Lv, Wenhao Li, Guohua Huang, Zhihui Hu, Jianmei Xu

**Affiliations:** 1 Faculty of Materials Science and Chemistry, China University of Geosciences, Wuhan 430074, China; hetiancheng@cug.edu.cn (T.H.); yx126523@126.com (C.L.); lwh3053@cug.edu.cn (W.L.); MH202011@cug.edu.cn (Z.H.); 2 Key Laboratory of New Electric Functional Materials of Guangxi Colleges and Universities, Nanning Normal University, Nanning 530023, China; hgh9@263.net

**Keywords:** microwave dielectric ceramics, Ba_6−3*x*_(Sm_1−*y*_Nd*_y_*)_8+2*x*_Ti_18_O_54_ (*x =* 2/3) system, dielectric constant, linear regression analysis

## Abstract

The electronics related to the fifth generation mobile communication technology (5G) are projected to possess significant market potential. High dielectric constant microwave ceramics used as filters and resonators in 5G have thus attracted great attention. The Ba_6−3*x*_(Sm_1−*y*_Nd*_y_*)_8+2*x*_Ti_18_O_54_ (*x =* 2/3) ceramic system has aroused people’s interest due to its underlying excellent microwave dielectric properties. In this paper, the relationships between the dielectric constant, Nd-doped content, sintering temperature and the density of Ba_6−3*x*_(Sm_1−*y*_Nd*_y_*)_8+2*x*_Ti_18_O_54_ (*x =* 2/3) ceramics were studied. The linear regression equation was established by statistical product and service solution (SPSS) data analysis software, and the factors affecting the dielectric constant have been analyzed by using the enter and stepwise methods, respectively. It is found that the model established by the stepwise method is practically significant with *Y* = −71.168 + 6.946*x*_1_ + 25.799*x*_3_, where *Y*, *x*_1_ and *x*_3_ represent the dielectric constant, Nd content and the density, respectively. According to this model, the influence of density on the dielectric constant is greater than that of Nd doping concentration. We bring the linear regression analysis method into the research field of microwave dielectric ceramics, hoping to provide an instructive for the optimization of ceramic technology.

## 1. Introduction

With the development of mobile communication equipment and portable terminals towards miniaturization and multifunctionality, the demand for filters and resonators have been increasing steadily. Microwave dielectric ceramics are the key materials of filters and resonators for telecommunications [1,2,3,4,5]. There are three important parameters to evaluate the ceramics: dielectric constant *ε_r_*, quality factor *Qf* and temperature coefficient of resonate frequency *τ_f_*. High dielectric constant is beneficial for microwave devices because the size of dielectrics is inversely proportional to $\sqrt{\varepsilon_{r}}$. Currently, BaO-Ln_2_O_3_-TiO_2_ (Ln: lanthanide) based systems exhibit superior properties in the high dielectric materials, which have aroused considerable attention [6,7,8,9]. For the solid solution of Ba_6−3*x*_(Sm_1−*y*_Nd*_y_*)_8+2*x*_Ti_18_O_54_, it has a like-perovskite tungsten bronze structure with TiO_6_ octahedra. According to the atomic occupation, the structural formula of the solid solution can be written into [Ln_8+2_*_x_*Ba_2−3_*_x_*V*_x_*]_A1_[Ba_4_]_A2_Ti_18_O_54_ (0 ≤ *x* ≤ 2/3, V:Vacancy). A1 is the rhombic site and A2 is the pentagonal site [10,11]. Suzuki et al. attributed a significant drop in *Qf* to the occupation of large Ba by Sr at the A1-site [12]. Amaral et al. demonstrated that there exists evident crystal orientation in thick films, which controls the microwave dielectric properties of the ceramics [13]. Vilarinho et al. obtained different dielectric constants by adjusting the thickness of BaLa_2_Ti_4_O_15_ and Ba_4_Nd_9.33_Ti_18_O_54_ films [14]. Wang et al. investigated the process and microwave dielectric properties of the BaTiO_3_ and BaTe_4_O_9_ system, and especially explored systematically the relationship between the microwave dielectric properties and Nd/Sm ratio and *x* value of Ba_6−3*x*_(Sm_1−*y*_Nd*_y_*)_8+2*x*_Ti_18_O_54_ [15,16,17,18]. According to previous studies [19], Ba_6−3*x*_Sm_8+2*x*_Ti_18_O_54_ ceramic has the best comprehensive properties, for example, high dielectric constant, high quality factor and negative resonant frequency temperature coefficient (*τ_f_*), while Ba_6−3*x*_Nd_8+2*x*_Ti_18_O_54_ ceramic presents a positive *τ_f_*. Therefore, the co-doping of Sm and Nd in the Ba_6−3*x*_(Sm_1−*y*_Nd*_y_*)_8+2*x*_Ti_18_O_54_ (*x =* 2/3) system would lead to near zero *τ_f_*, which is very important to improve the frequency stability of the devices. Although the effect of co-doping of Sm and Nd on the properties of Ba_6−3*x*_(Sm_1−*y*_Nd*_y_*)_8+2*x*_Ti_18_O_54_ has been investigated [20,21], the statistical analysis method was rarely used to study the factors affecting the performance.

Statistical product and service solutions (SPSS) are mathematical analysis software, which include the correlation analysis, regression analysis, cluster analysis, discriminant analysis, etc. The regression analysis can be divided into linear regression analysis, non-linear regression analysis, curve estimation and logistic regression. Among them, linear regression analysis is especially valuable in determining the critical factor [22,23]. Doreswamy proposed the linear regression model and predicated the attribute values from the large engineering materials database [24]. The study of regression analysis suggested that data mining techniques is applicable to the investigation on material informatics, which could facilitate the development of new materials. For microwave dielectric ceramics, there are many factors to influence the dielectric constant, for example, element doping, sintering temperature and density. SPSS is thus employed to determine the critical factor of affecting the dielectric constant.

There are many different methods to establish mathematical models by SPSS, such as the enter method (forced entry method), the forward method, the backward method and the stepwise method. The enter method can generate rich information via taking all the factors into consideration, but its sophisticated nature introduces disputes on the results. The stepwise method combines the merits of the forward and backward methods and can establish the mathematical model with fast speed. Consequently, in this paper, we applied the enter and stepwise methods to create the mathematical models and analyze the factors that affect the dielectric constant of Ba_6−3*x*_(Sm_1−*y*_Nd*_y_*)_8+2*x*_Ti_18_O_54_ (*x =* 2/3) microwave dielectric ceramics. The relationships between the dielectric constant, Nd dopant, sintering temperature and the density have been established by using the models, which is valuable for the development of high-performance BaO-Ln_2_O_3_-TiO_2_ dielectric ceramics for microwave communication applications.

## 2. Experimental Process

### 2.1. Materials

The starting materials including BaCO_3_ (99.0%), Sm_2_O_3_ (99.9%), Nd_2_O_3_ (99.9%) and TiO_2_ (99.0%) were weighed in stoichiometric ratios according to the chemical formula of Ba_6−3*x*_(Sm_1−*y*_Nd*_y_*)_8+2*x*_Ti_18_O_54_ (*x =* 2/3) with *y* = 0, 0.1, 0.2, 0.3 and 0.4.

### 2.2. Preparation of Ceramics

The ceramics were made from the starting materials, which were mixed and grounded for 4 h with ethanol using polyurethane jars and zirconia balls by the Planetary Ball Mill (Hunan Future Mechanical & Equipment Manufacturing CO., LTD, Yueyang, China), then dried and calcined at 1050 °C for 2 h. The furnace type is KSL-1750X-S (made in Hefei Kejing Materials Technology CO., LTD, Hefei, China). The calcining powders with 5 wt % PVA (from Aladdin Holdings Group, Beijing, China) binder were passed through a mesh and pressed into pellets with a 2:1 ratio of their diameters to thicknesses. The sizes of samples were 10 mm in diameter and 5 mm in thickness. These pellets were heat treated at 550 °C for 1 h with 5 °C/min of heating rate, and then sintered in air at 1300 °C, 1350 °C and 1400 °C for 2 h respectively with 10 °C/min of heating rate. When the heating schedule was finished, it was cooled with the furnace.

### 2.3. Ceramics Characterization

The crystal structures of the ceramics were characterized by *X*-ray diffraction using a Bruker AXS D8-Focus diffractometer (Bruker Corporation, Karlsruhe, Germany) with Cu *Kα* radiation (λ = 1.540598 Å). The step size was 0.02° and the scan rate was 5°/min. The densities were tested by the DE-200M apparatus made by HONGTUO Instrument (Dongguan, China). The measurement method of ceramic density was based on the Archimedes principle. The immersion liquid was the water. The formula for calculating the bulk density of the sample is according to Equation (1).

*ρ* = *m*_0_ × *ρ_w_*/(*m*_1_ − *m*_2_) (1)

*ρ* is bulk density of the ceramic, g/cm^3^; *ρ_w_* is the density of the water at the temperature of the test, g/cm^3^; *m*_0_ is the weight of the ceramic after drying at 105 °C for 3 h, g; *m*_1_ is the weight of the ceramic in air with water saturation after vacuum-pumping, g, and *m*_2_ is the weight of the ceramic in water with water saturation after vacuum-pumping, g.

The morphologies of the ceramics were observed with a scanning electron microscope (SEM, SU8010, Hitachi, Tokyo, Japan). The microwave dielectric properties were measured using the Hakki and Coleman method by the Agilent E8362B network vector analyzer (Agilent, Palo Alto, USA). The spectroscopy frequency range is from 10 MHz to 20 GHz, and the resonant frequencies of all samples made in our study were lower than 10 GHz. The temperature range was from 20 to 80 °C for determining the temperature coefficient of the resonance frequency *τ_f_*.

## 3. Results and Discussion

### 3.1. X-ray Characterization of Ba_6−3x_(Sm_1−y_Nd_y_)_8+2x_Ti_18_O_54_ (x = 2/3) Ceramics

**Figure 1:**
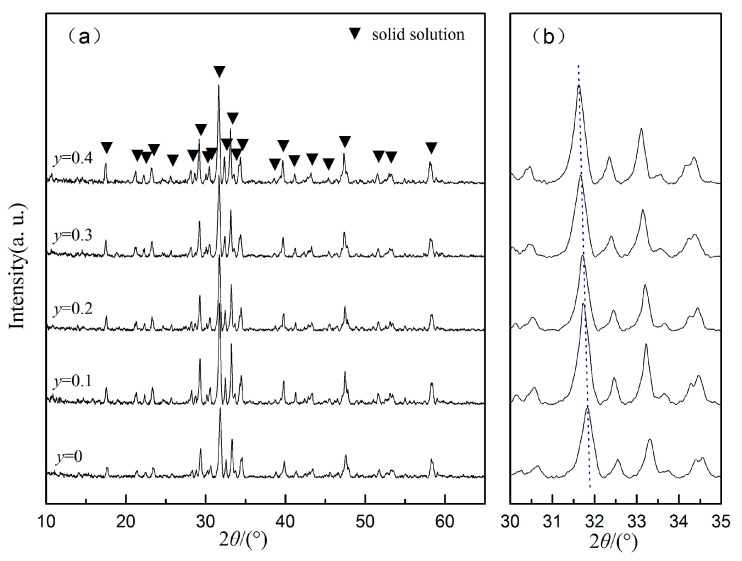
XRD diffraction patterns of Ba_6−3*x*_(Sm_1−*y*_Nd*_y_*)_8+2*x*_Ti_18_O_54_ (*x* = 2/3, *y* = 0, 0.1, 0.2, 0.3, 0.4) ceramics sintered at 1400 °C for 2 h: (**a**) patterns for 2*θ* from 10 to 65° and (**b**) partial enlarged detail of XRD patterns around 2*θ* = 31.7°.

*X*-ray diffraction patterns of Ba_6−3*x*_(Sm_1−*y*_Nd*_y_*)_8+2*x*_Ti_18_O_54_ (*x =* 2/3) ceramics sintered at 1400 °C for 2 h are illustrated in Figure 1. According to the XRD standard card PDF #43-0235, the ceramics were pure phase Ba_6−3*x*_(Sm_1−*y*_Nd*_y_*)_8+2*x*_Ti_18_O_54_ (*x =* 2/3) solid solution with Nd contents ranging from *y* = 0 to 0.4. Figure 1b shows that the peaks at the vicinity of 2*θ* = 31.7° shifted towards lower degrees with the increase of Nd content, which suggests that Sm ions (*r* = 1.098 Å with hexa-coordinate [25]) were replaced by Nd ions with a larger radius (*r* = 1.123 Å with hexa-coordinate [25]) [15]. The ceramics show the properties of pure Ba_6−3*x*_(Sm_1−*y*_Nd*_y_*)_8+2*x*_Ti_18_O_54_ (*x =* 2/3) solid solution phases without impurities.

### 3.2. Establishment of the Mathematical Models Based on the SPSS Multiple Linear Regression Analysis

The resonate frequency *f*, dielectric constant *ε_r_* and quality factor *Qf* were measured at microwave frequencies by the Hakki and Coleman method. For the specific ceramics, the resonate frequency is constant, so the dielectric constant value is very reliable. The mathematical models of the dielectric constant were established, in which *Y*, *x*_1_, *x*_2_ and *x*_3_ denote the dielectric constant, Nd content relative to Sm, sintering temperature (°C) and the density (g/cm^3^), respectively. Table 1 summarizes the densities and dielectric constants of the ceramics with different Nd contents and sintered at different temperatures. The Nd contents *x*_1_ varied from 0 to 0.4, while the sintering temperatures *x*_2_ from 1300 to 1400 °C at which the samples were fired into ceramics. According to Table 1, the multiple linear regression mathematical models were established by using SPSS software.

### 3.3. Linear Regression Model Based on the Enter Method

The enter method is also named as the forced enter method, while all of the arguments are introduced into the model simultaneously. Generally, the enter method is suitable to find out the significance of the argument. By fitting the enter method, the arguments that are nonsignificant will be found.

Table 2 is the summary of the regression model based on the enter model. The parameters were obtained with the correlation coefficient *R* = 0.973 and coefficient of determination *R*^2^ = 0.947. *R*^2^ refers to the fitting degree, which indicates the quality of the model. The closer *R*^2^ is to 1, the more appropriate the model will be. The adjusted multiple correlation coefficient *R_a_*^2^ is 0.943, which confirms that there are strong linear correlations between dielectric constants and Nd content, sintering temperature and the density of the ceramics [26]. The *F*-value (joint hypotheses test) was 243.722, and *p*-value (probability value under the corresponding *F*-value) was 0.000, which further confirmed that our model was suitable. The *p*-value was smaller than 0.05, which indicates that the linear regression equation had passed the 0.05 alpha-level significance test. From Table 2, the Durbin–Watson test value (*DW*) was 2.112, which means that the model does not have self-correlation [27].

The regression equation (Equation (2)) can be obtained by the regression coefficient shown in Table 3:

*Y* = −73.375 + 7.347*x*_1_ + 0.107*x*_2_ + 23.568*x*_3_ (2)

According to the regression Equation (2), when the density increases by 1 g/cm^3^, the dielectric constant will increase by 23.568. When the sintering temperature increases by 100 °C, the dielectric constant will increase by 10.7. When Nd content is increased by 1% relative to that of Sm, the dielectric constant will enhance by 0.07347.

Both the arguments *x*_1_ and *x*_3_ have passed the significance test for the confidence level *α* = 0.05. However, the *p*-value for *x*_2_ was 0.073, which is greater than 0.05. This suggests that *x*_2_ should not be an argument in this linear regression model, which will be corroborated by the stepwise regression method as shown in our following discussion. From Equation (2), the most important impact factor on *Y* is *x*_3_, indicating that the density is the most important parameter affecting the dielectric constant. The relationship between densities and dielectric constants is also verified from Table 1. It is found that density plays a more important role than temperature. In fact, the density is related to sintering temperature. When the temperature was lower than the optimized sintering temperature range, the density gradually increased to the maximum value with increasing temperature. While the temperature was beyond the optimized sintering temperature range, the ceramics were over-fired, and consequently, the densities decreased. However, in this model, there were no significance correlations between temperatures and dielectric constants. We thought that the impact of temperatures might be included in the density factor. If fired at lower temperatures, the ceramics will not be densified and deteriorate the densities. Therefore, temperature and density should not be considered concurrently as influencing factors. From Table 3, the VIFs (variance inflation factor) of three arguments *x*_1_, *x*_2_ and *x*_3_ were less than 10, which suggests that there was no multicollinearity among them [28,29].

In Table 3, Beta is the standardization coefficient and *B* is the non standardization coefficient. In SPSS, the standardization is to eliminate the error caused by different units among arguments and dependent variable. The data standardization method is that the value of original data subtracts the mean of the corresponding variable and then divides by the standard deviation of the variable. The calculated regression equation is called the standardized regression equation, and the corresponding regression coefficient is the standardized regression coefficient. The relationship between the non standardized coefficient *B* and standardized coefficient Beta can be expressed by Equation (3):

Beta = *B* × σ*_x_*/σ*_y_* (3)

Beta is the standardized coefficient; *B* is the non standardized coefficient; *σ_x_* is the standard deviation of an argument and *σ_y_* is the standard deviation of the dependent variable.

The standardized coefficient can be used to evaluate which one is more important among all arguments. The non standardized coefficient reflected the absolute effect of the change of an argument on the dependent variable. From Table 3, the maximum Beta was 0.827 for *x*_3_, so among *x*_1_, *x*_2_ and *x*_3_, *x*_3_ was the most important for the dielectric constant.

**Figure 2:**
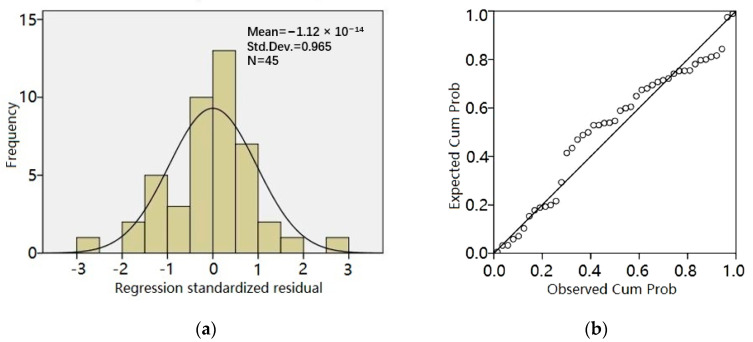
The results based on the enter method: (**a**) the residual frequency distribution histogram and (**b**) the residual normal probability plot.

Figure 2 is the residual frequency distribution histogram and residual normal probability plot based on the enter method. Figure 2a is the normal distribution, and the dots were nearly on a straight line in Figure 2b, which shows that the mathematical model established by the enter method had passed the error test of normality. Through the Shapiro–Wilk test, the level of significance was more than 0.05, so this further proved the model was suitable.

### 3.4. Linear Regression Model Based on the Stepwise Method

The stepwise method is totally different from the enter method. In the stepwise method, the arguments are introduced into the model one by one. The *F*-value test was performed at each step to ensure that only significant arguments were included in the regression equation before the new argument was introduced. Table 4 is the summary of the regression model based on the stepwise method. Model 1 is the transition model and Model 2 is the final model. In the process of simulation, the *x*_3_ argument was firstly chosen by the software, leading to the strongest linear correlation between *x*_3_ and *Y*. After the *x*_1_ argument was chosen, the linear regression model was established between *Y* and *x*_1_ and *x*_3_, while *x*_2_ was eliminated. For the mathematical model established by the enter method, the argument *x*_2_ did not pass the test of normality. For the model established by the stepwise method, *x*_2_ was eliminated and not introduced into the model. As mentioned above, as the impact of the temperature on the dielectric constant has already been included in the density, we did not need to consider the influence of both temperature and density simultaneously.

For Model 2, *R_a_*^2^ was 0.940, which made the linear fitting result better than Model 1. The statistical significance for *p*-value of the model was 0.000, which passed the test of confidence level α = 0.05. Model 2 had no self-correlation for *DW* = 2.184, and passed the error independence test. For Model 1, it had also no self-correlation. Since there was only one argument, the *DW* was zero.

From Table 5, the linear regression model can be obtained by the stepwise regression method as follow (Equation (4)):

*Y*= −71.168 + 6.946*x*_1_ + 25.799*x*_3_, (4)

The probability value *p*-value of both *x*_1_ and *x*_3_ were 0.000, which shows that the model had passed the significance test and the arguments *x*_1_ and *x*_3_ had statistical significance. The *VIF* values were 1.029 for *x*_1_ and *x*_3_, and there was no multicollinearity between the arguments. Comparing the Beta value, it was found that the Beta value (0.906) of *x*_3_ was much greater than that (0.229) of *x*_1_, which implies that *x*_3_ was more important than *x*_1_.

**Figure 3:**
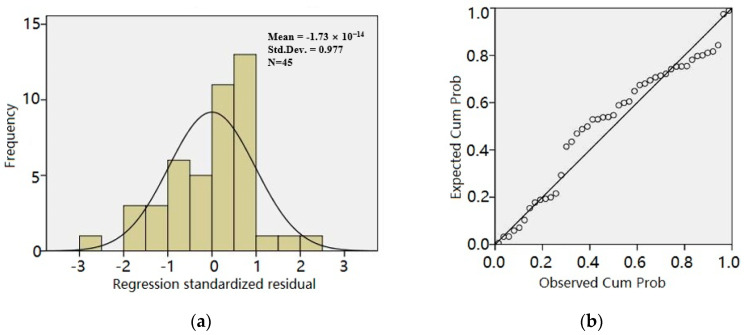
Normal *p–p* plot of regression standardized residual based on the stepwise method: (**a**) residual frequency distribution histogram and (**b**) residual normal probability plot.

Figure 3 is the residual frequency distribution histogram and residual normal probability plot obtained by the stepwise method. As shown in Figure 3a, the residual frequency distribution histogram met the normal distribution. Figure 3b shows that almost all the points were on a line. By the Shapiro–Wilk test, the level of significance was more than 0.05. We thus concluded that the hypothesis about the error normality was reasonable and the model passed the test of the error normality.

### 3.5. Comparison of Two Regression Models

For more than one argument, adjusted multiple correlation coefficient *R_a_*^2^ can be used to evaluate the quality of the model. The closer the value is to 1, the better the model is. In this study, *R_a_*^2^ were 0.943 and 0.940 for the enter regression model and the stepwise model, respectively. Therefore, both models were of high quality and could be used to explain the relationship between the dependent variable and the arguments. For the enter method, the argument *x*_2_ did not pass the significance test, which shows that temperature had little effect on the dielectric constant. As mentioned above, because there were close relationships between temperature and the density, it was not appropriate for them to be considered as impact factors at the same time. For Model 2 established by the stepwise method without *x*_2_, although it includes fewer arguments without all the information, it meets the purpose that the reasonable, simply and useful regression models should be established with the most suitable and the least arguments [27].

### 3.6. Guidance of the Model on Other Properties of Microwave Dielectric Ceramics

**Figure 4:**
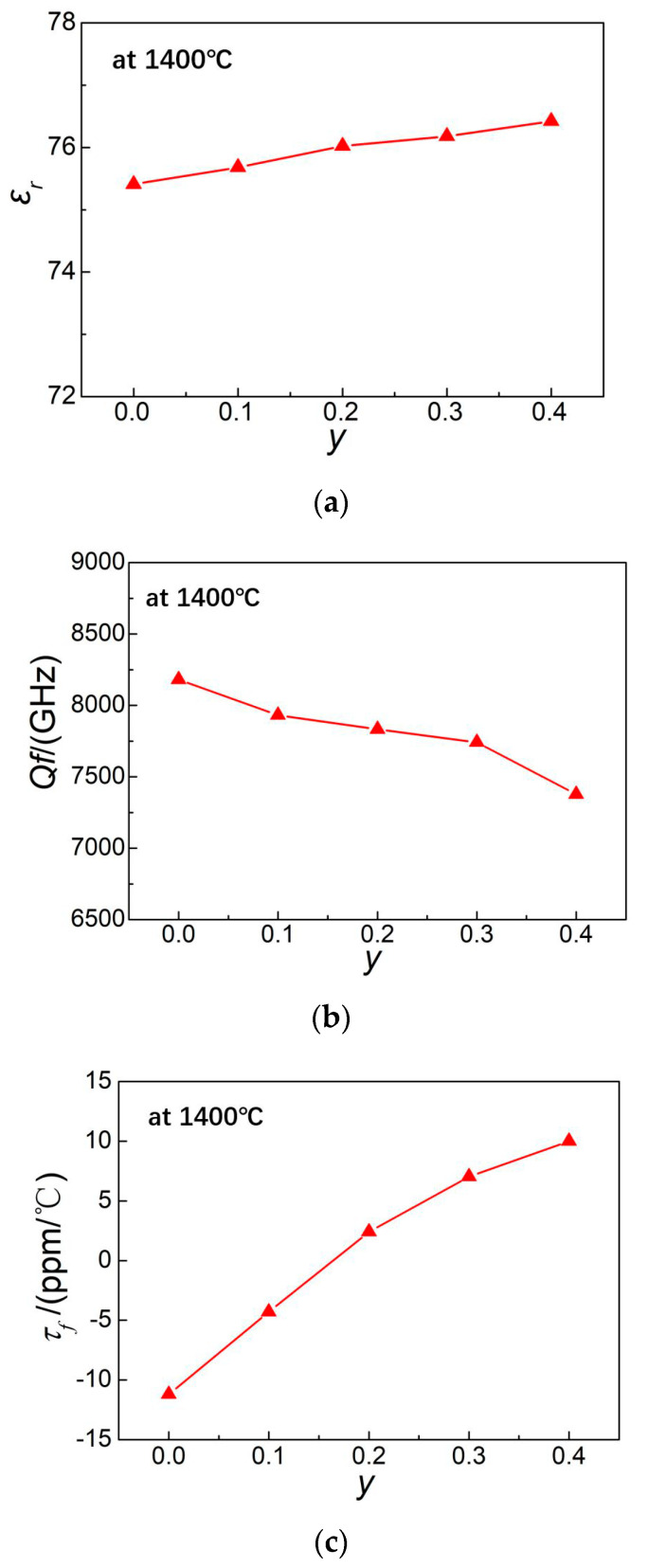
The relationship between Nd content and microwave dielectric properties: (**a**) dielectric constant; (**b**) quality factor and (**c**) temperature coefficient of Ba_6−3*x*_(Sm_1−*y*_Nd*_y_*)_8+2*x*_Ti_18_O_54_ (*x* = 2/3) ceramics sintered at 1400 °C.

For microwave dielectric ceramics there are three important performances (1) dielectric constant *ε_r_*, (2) quality factor *Qf* and (3) the temperature coefficient of resonant frequency *τ_f_*. For the BaO-Ln_2_O_3_-TiO_2_ system, there was a generally recognized correlation between the three performances, that is the temperature coefficient *τ_f_* was positively correlated and quality factor *Qf* was negatively correlated with the dielectric constant, which had been confirmed by many researches [19,21,30,31]. In this study, the same correlations among the three performances were also obtained, when referring to Figure 4. While *y* (i.e., molar content of Nd to Sm) from 0 to 0.4, *ε_r_* and *τ_f_* increased and *Qf* decreased with *y*. The suitable model was established for evaluating the effect of process parameters on dielectric constant by linear regression analysis. So, the effect of the same parameters on the quality factor and temperature coefficient could be estimated by the established model. According to the stepwise model, there was a positive correlation between Nd content and the dielectric constant, so there was also a positive correlation between Nd content and *τ_f_*, but a negative correlation for *Qf*. Due to the small number of samples, there was no linear regression analysis on the quality factor and temperature coefficient of ceramics in this study. Especially for the temperature coefficient test, it would take a lot of time to get enough data. In the future work, we will further study *Qf* and *τ_f_* by linear regression analysis.

**Figure 5:**
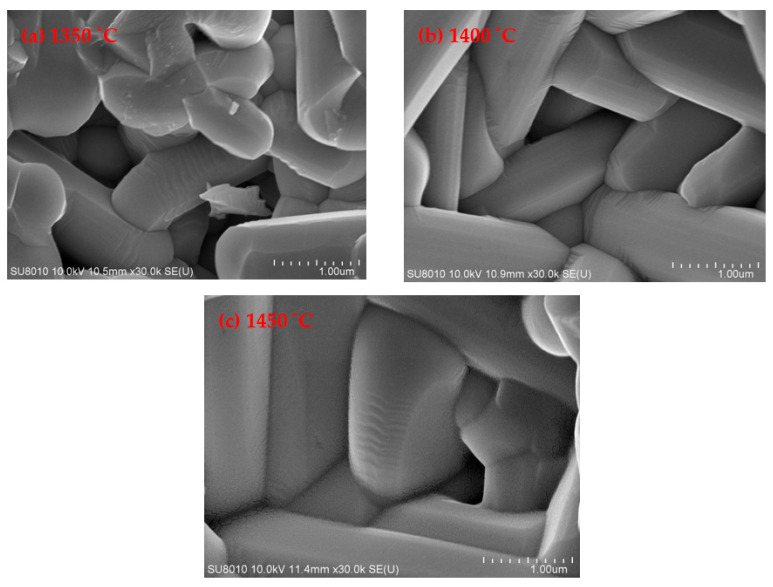
SEM morphologies of ceramics sintered at different temperature: (**a**) 1350 °C; (**b**) 1400 °C and (**c**) 1450 °C.

Figure 4 is the microwave dielectric properties of ceramics sintered at 1400 °C with different Nd content. At 1400 °C, the ceramics showed better properties than those sintered at other temperature. The scanning electron microscopes (SEMs) are illustrated in Figure 5. From Figure 5a, the ceramics sintered at 1350 °C appeared as incomplete grain growth, while the ceramics at 1450 °C (see Figure 5c) show overheated and some grains were melted together, which had the tendency of secondary grain growth who led to the enlargement of internal pores. However, the ceramics sintered at 1400 °C present grains with complete growth and uniform size. So, only the properties of ceramics sintered at 1400 °C were chosen to be discussed. As shown in Figure 4a, with the increase of Nd content, the dielectric constants of ceramics increased gradually, which was due to the contributions of cell volume and ionic polarizability of Nd dopant. Ba_6−3*x*_(Sm_1−*y*_Nd*_y_*)_8+2*x*_Ti_18_O_54_ (*x* = 2/3) solid solution is the like-perovskite tungsten bronze structure with the ABO_3_ type. The superlattice exists in the direction of c axis, which is due to the tilting of the titanium-oxygen octahedron [32]. The size and filling degree of ions at the A site will affect the tilt degree of Ti—O octahedron, and then change the cell volume. Nd dopant will occupy the A site, so the substitution of Nd for Sm affects the tilt degree of octahedron and cell volume. According to Clausius–Mossotti Equation (5), the dielectric constant of ceramics is determined by the cell volume *V_m_* and the total ionic polarizability α_D_, namely [26]:

(5) $$\varepsilon_{r}=\frac{3V_{m}+8\pi \alpha_{D}}{3V_{m}-4\pi \alpha_{D}},$$

The ionic polarizabilities of Nd^3+^ and Sm^3+^ were 5.01 Å^3^ and 4.74 Å^3^ respectively. So, the substitution of Nd^3+^ at A site influences not only the cell volume *V_m_*, but also the total ionic polarizability *α*_D_, which resulted in the change of dielectric constant *ε_r_*. The dopant element Nd with a large ionic radius and large ionic polarizability relative to Sm will increase the unit cell volume and then lead to the enlargement of the octahedral B site occupied by Ti^4+^ in the TiO_6_ octahedron, which accounted for the increases of the ionic electronic polarizability and then the dielectric constant. This was also confirmed by Wang et al. [15] and Valant et al. [33]. The dielectric constant is the synergistic effect of the unit cell volume and polarizability. Similarly, the lanthanum (La) element dopant with a large ionic radius of 1.36 Å and ionic polarizability of 6.03 Å^3^ will also increase the dielectric constant of ceramics, but decrease the quality factor. The influence of the La dopant will be reported elsewhere. This study will benefit the prediction of the dielectric constant of ceramics with different dopants and guide the investigation of other ceramic systems.

## 4. Conclusions

Microwave dielectric ceramics with high dielectric constants were prepared. The two kinds of linear regression models were established and compared. The model built by the stepwise method had more practical significance than that by the enter method. The better model was *Y* = −71.168 + 6.946*x*_1_ + 25.799*x*_3_, where *Y*, *x*_1_ and *x*_3_ represent the dielectric constant, Nd content and the density, respectively, which disclosed the relationship between the dielectric constants and Nd content and the density of Ba_6−3*x*_(Sm_1−*y*_Nd*_y_*)_8+2*x*_Ti_18_O_54_ (*x* = 2/3) microwave dielectric ceramics. Based on the model, it is clear that the density (*x*_3_) was more important to the dielectric constant (*Y*) than Nd content (*x*_1_), owing to the standardized coefficient Beta value that was 0.906 for *x*_3_ but 0.229 for *x*_1_, while a large Beta value indicates that it was more significant. This model will help to instruct how to optimize the preparation technology in order to obtain high dielectric constant microwave ceramics.

## Figures and Tables

**Table 1:** Densities and dielectric constants with different Nd contents and sintering temperatures.

*Y*	*x*_1_	*x*_2_ (°C)	*x*_3_ (g/cm^3^)
61.83	0	1300	5.18
73.03	0	1350	5.62
75.55	0	1400	5.65
63.12	0.1	1300	5.17
74.35	0.1	1350	5.61
75.68	0.1	1400	5.64
71.51	0.2	1300	5.46
73.30	0.2	1350	5.61
75.28	0.2	1400	5.61
72.25	0.3	1300	5.47
74.97	0.3	1350	5.59
75.97	0.3	1400	5.61
72.21	0.4	1300	5.44
74.20	0.4	1350	5.56
76.34	0.4	1400	5.59

Notes: *Y*—Dielectric constant; *x*_1_—Nd content; *x*_2_—Sintering temperature; *x*_3_—Density.

**Table 2:** Summary of the regression model based on the enter model.

Model	*R*	*R* ^2^	*R_a_* ^2^	*SEE*	*F*-Value	*p*-Value	*DW*
1	0.973	0.947	0.943	1.03618	243.722	0.000	2.112

Notes: *R*—Correlation coefficient; *R*^2^—Coefficient of determination; *R_a_*^2^—Adjusted multiple coefficient of determination; *SEE*—Estimated standard error; *F*-value—Joint hypotheses test (the total significance test for regression equation); *p*-value—Probability value under the corresponding *F*-value; *DW*—Durbin–Watson test value.

**Table 3:** Regression coefficients based on the enter model.

Models	Non Standardized Coefficient		Standardized Coefficient	*t*	*p*-Value	*VIF*
	*B*	Standard Error	Beta			
1 (Constant)	−73.375	5.834	-	−12.576	0.000	-
*x*_1_	7.347	1.129	0.242	6.506	0.000	1.069
*x*_2_	0.107	0.058	0.102	1.842	0.073	2.356
*x*_3_	23.568	1.596	0.827	14.763	0.000	2.425

Notes: *B*—Non standard regression coefficient; Beta—Standard regression coefficient; *t*—Hypothesis test of partial regression coefficient; *p*-value—Probability value under the corresponding *F* value; *VIF*—Variance inflation factor (when it is larger than 10, there is serious multicollinearity).

**Table 4:** Summary of the regression model based on the stepwise method.

Models	*R*	*R* ^2^	*R_a_* ^2^	*SEE*	*F-*Value	*p-*Value	*DW*
Model 1	0.944	0.892	0.889	1.44551	353.77	0.000	-
Model 2	0.971	0.943	0.940	1.06529	344.272	0.000	2.184

Notes: *R*—Correlation coefficient; *R*^2^—Coefficient of determination; *R_a_*^2^—Adjusted multiple coefficient of determination; *SEE*—Estimated standard error; *F*-value—Joint hypotheses test (the total significance test for regression equation); *p*-value—Probability value under the corresponding *F* value; *DW*—Durbin–Watson test value; Model 1—only one argument *x*_3_; Model 2—two arguments *x*_3_ and *x*_1._

**Table 5:** Regression coefficient based on the stepwise model.

Model	Non Standardized Coefficient		Standardized Coefficient	*t*	*p*-Value	*VIF*
	*B*	Standard Error	Beta			
1 (Constant)	−75.847	7.897	-	−9.604	0.000	-
*x*_3_	26.899	1.430	0.944	18.809	0.000	1.000
2 (Constant)	−71.168	5.871	-	−12.123	0.000	-
*x*_3_	25.799	1.069	0.906	24.128	0.000	1.029
*x*_1_	6.946	1.139	0.229	6.097	0.000	1.029

Notes: *B*—Non standard regression coefficient; Beta—Standard regression coefficient; *t*—Statistics for test; *p*-value—Probability value under the corresponding *F* value; *VIF*—Variance inflation factor (when it is larger than 10, there is serous multicollinearity); *x*_3_—Density; *x*_1_—Nd content.
